# Mast Cell Activation Syndrome Update—A Dermatological Perspective

**DOI:** 10.3390/jpm13071116

**Published:** 2023-07-10

**Authors:** Dana Mihaela Mihele, Paul Andrei Nistor, Gabriela Bruma, Cristina Iulia Mitran, Madalina Irina Mitran, Carmen Elena Condrat, Mihaela Tovaru, Mircea Tampa, Simona Roxana Georgescu

**Affiliations:** 1 Dermatology Department, Carol Davila University of Medicine and Pharmacy, 8 Eroii Sanitari Blvd, 050474 Bucharest, Romania; dmihele@yahoo.com (D.M.M.); mihaelatovaru@yahoo.fr (M.T.); srg.dermatology@gmail.com (S.R.G.); 2 Dermatology Department, Victor Babes Clinical Hospital of Infectious and Tropical Diseases, 030303 Bucharest, Romania; gabriela.bruma@yahoo.com; 3 Internal Medicine Department, Emergency University Hospital Bucharest, 169 Independence Blvd, 050098 Bucharest, Romania; paul.nistor@outlook.com; 4 Microbiology Department, Carol Davila University of Medicine and Pharmacy, 8 Eroii Sanitari Blvd, 050474 Bucharest, Romania; madalina.irina.mitran@gmail.com; 5 Fetal Medicine Excellence Research Center, Alessandrescu-Rusescu National Institute for Mother and Child Health, 020395 Bucharest, Romania; drcarmencondrat@gmail.com; 6 Department of Obstetrics and Gynecology, Carol Davila University of Medicine and Pharmacy, 8 Eroii Sanitari Blvd, 050474 Bucharest, Romania

**Keywords:** mast cells, MCAS, dermatology, anaphylaxis, systemic

## Abstract

Mast cells (MCs) are infamous for their role in potentially fatal anaphylaxis reactions. In the last two decades, a more complex picture has emerged, as it has become obvious that MCs are much more than just IgE effectors of anaphylaxis. MCs are defenders against a host of infectious and toxic aggressions (their interactions with other components of the immune system are not yet fully understood) and after the insult has ended, MCs continue to play a role in inflammation regulation and tissue repair. Unfortunately, MC involvement in pathology is also significant. Apart from their role in allergies, MCs can proliferate clonally to produce systemic mastocytosis. They have also been implicated in excessive fibrosis, keloid scaring, graft rejection and chronic inflammation, especially at the level of the skin and gut. In recent years, the term MC activation syndrome (MCAS) was proposed to account for symptoms caused by MC activation, and clear diagnostic criteria have been defined. However, not all authors agree with these criteria, as some find them too restrictive, potentially leaving much of the MC-related pathology unaccounted for. Here, we review the current knowledge on the physiological and pathological roles of MCs, with a dermatological emphasis, and discuss the MCAS classification.

## 1. Introduction

Mast cells (MCs) have received constant attention since their contribution to allergic reaction was uncovered, yet they remain one of the least understood elements of the immune system. Most MC research has focused on their pathological disturbances while their physiological role has been all but ignored. A reader can hardly consult any MC-related article without being told, usually in the first paragraph, that these cells are responsible for potentially lethal IgE-mediated anaphylactic shock. This view of MCs as villains of the immune system has been thus described in a review: “The existence of these potentially hazardous cells has solely been justified due to their beneficial role in some infections with extracellular parasites” [1]. However, a significant body of data, produced largely in the last two decades, has revealed a plethora of functions ranging from defense against infections and toxins to immune surveillance and homeostasis. More recently, expressing the view of a complex role of MCs, Stephen Galli stated: “We do not have mast cells and IgE so that we can eat peanut and die!” [2]. MCs’ role as “protectors of health” has been recently reviewed [3].

## 2. Mast Cell Basic Physiology

Mast cells are long-lived, tissue-residing granulocytes that originate in the bone marrow. They share a common myeloid precursor with other granulocytes, including their circulating counterpart, the basophils. Unlike other granulocytes, MCs leave the bone marrow as progenitors fated to complete their maturation in their tissue of residence where they can further expand upon appropriate stimulation [4]. Importantly, they return to baseline numbers in the region of interest when the inflammatory stimulus has subsided [5].

MC tissue distribution follows their role as cells of first defense against potential aggressions. They are present in higher numbers in zones of contact with exogenic antigens, such as skin and cavitary organs. In addition, they concentrate around blood vessels in virtually all tissues [6]. There, MCs are classically activated by IgE antibodies through the high affinity IgE receptors (FcεRIs), which in humans are present on the surface of MCs, basophils and eosinophils [7]. Less efficiently, but probably of greater importance for their function, MCs can be activated through the IgG receptors, cytokines and directly by antigens of invading organisms, the best documented of which are bacteria and parasites [8].

**Figure 1:**
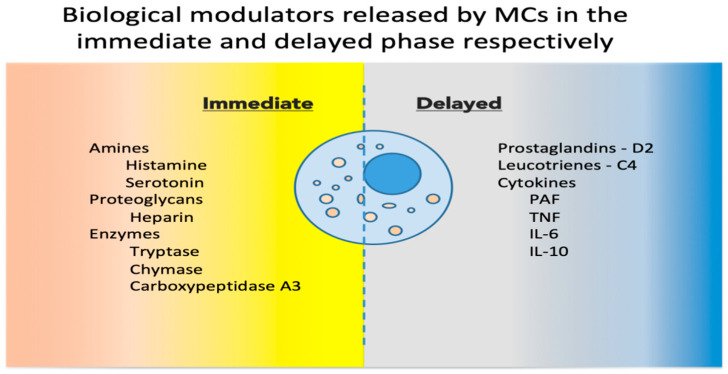
Biological modulators released by MCs in the immediate and delayed phases [12,13]. MCs, mast cells; PAF, platelet activating factor; TNF, tumor necrosis factor; IL, interleukin.

Upon activation, MCs release a host of mediators involved in local defense and homeostasis. Their response has been described as biphasic: immediate—in minutes, preformed mediators are released from granules (degranulation), and delayed—in hours, biological modulators are actively secreted [9]. See Figure 1 for a synthetic list of released and secreted molecules. MC heterogeneity has been described in relation to the biochemical components of their granules, and historically, based on the two main MC proteases (tryptase and chymase), MCs have been classified into MC_T and MC_TC, containing only tryptase, or both tryptase and chymase, respectively [10]. These populations, and others discovered since, are now considered reversible phenotypes acquired in response to environmental cues [11]. 

Tryptase and chymase are specific MC and basophil enzymes and can be used as biomarkers of inflammation. Tryptase is the most abundant MC enzyme and currently is the only standardized marker of MC activity, with a threshold of 11.4 ng/mL as the maximum accepted level in the serum of healthy individuals [14,15]. Tryptase has pleiotropic roles that are both protective and harmful [16]. Of chymase’s many actions, the one which elicits particular interest is its ability to convert angiotensin I to angiotensin II, and thus bypass the anti-hypertensive activity of the widely used angiotensin converting enzyme inhibitors [17]. 

In humans, tryptase is encoded by TPSAB (Tryptase Alpha/Beta) genes, of which there are normally four copies in the human genome. Variability was observed by Lyons et al., reporting that through gene duplication events, up to four extra copies can be found aggregated in families, and increased gene copies correlate with increased serum tryptase levels [18].

TPSAB gene heterogeneity may also involve loss of function mutations, and despite significant variability within human populations, variants with less than two copies have not been observed [19].

Increased tryptase serum levels are associated with mast cell activation syndrome, and this aggregates within families [20]. Interestingly, for a seemingly unrelated gene, CACNA1H, encoding for the Cav3.2 subunit of a calcium channel, three functional variants imparting partial gain of function have been reported. These variants frequently co-segregate with increased allelic TPSAB copies. Signaling through this calcium channel has been suggested to play a role in models of irritable bowel syndrome and histamine independent pruritus. In humans, gain of function is associated with hypertension and absence seizures in children [21].

## 3. Mast Cells as Immune Tissue Effectors

Strategically placed at the interface with the environment, MCs participate as the first line of organism defense [8,22]. As fast-activated immune effectors, they may often be the first resident cells to respond to an insult [3]. MCs not only initiate but also organize the immune response, recruiting multiple immune effectors, such as neutrophils [23,24], T cells [25] and NK cells [26,27], to the site of infection. Furthermore, MCs may enhance neutrophil effector functions through release of chemokines such as IL6, TNF and GM-CSF [28,29], and they further facilitate neutrophil and T cell recruitment through inflammatory vasodilatation, endothelium activation and connective tissue relaxation. [30]. MCs can modulate dendritic cell functions, promoting their migration, maturation and T cell priming capacity and influence in this way the adaptive immunity [27,31,32,33]. 

MCs also have, in some circumstances, antigen-presenting capacity [34,35]. They can modulate T cell functions through the release of soluble mediators and exosomes, orienting T cell polarization toward TH1, TH2 or TH17 and are able to directly inhibit effector T cells via IL-10 [36,37,38].

“MCs sense cell stress and tissue damage through cytokines, alarmins and purinergic receptors” [3], and detect infective agents indirectly through Fc and complement receptors directly through Toll-like receptors [39,40,41]. Once activated, MCs release their granules, which inhibit bacterial growth, are cytotoxic to helminths, and increase intestinal epithelial barrier permeability—a process which was implicated in increased parasite expulsion [41,42,43]. Moreover, MCs directly participate in bacterial killing by phagocytosis and release of antimicrobial peptides (e.g., lipocalin 2 and cathelicidin) and extracellular traps [44,45,46,47].

MC proteases have been shown to degrade harmful components of multiple venoms, including those from snakes, spiders, scorpions, bees and Gila monster [48,49,50]. Moreover, previous sensitization can, through IgE-dependent activation of MCs, enhance the likelihood of survival to potentially lethal doses of venom on a second challenge [51,52].

## 4. Role of Mast Cells in Pathology

However, MCs are not always the heroes. The list of grievances against these cells is long, and their implications in morbidity and mortality substantial, even ignoring their well-known role in IgE-mediated allergy and anaphylaxis reactions. 

For example, MCs have been implicated in excessive fibrosis and keloid scarring [53], although physiological roles in protection against excessive fibrosis have also been reported in certain contexts [54]. In kidney disease, the MC number increases many fold, and there they may contribute to vasoconstriction, on an already poorly vascularized kidney, through chymase-activated angiotensin II. MC presence has been associated with kidney loss of function and fibrosis. At this level, MCs have been shown to regulate inflammation, and both pro- and anti-inflammatory activity have been reported in diseased kidney [55,56]. Furthermore, MCs have been implicated in graft rejection [57], cancer progression [58], inflammatory bowel disease [59,60], atherosclerotic plaque progression [61], arthritis [62], osteoporosis [63], psoriasis [64,65,66,67], atopic [68,69] and allergic contact dermatitis [70,71,72].

Nevertheless, MCs’ contribution to immune-mediated pathology should not be overstated; at least some auto-immune diseases have been shown to progress unhindered by the absence of MCs [73]. 

A relationship between Ehlers–Danlos syndrome (EDS) and abnormal MC activation has been proposed [74], as well as a triple association: MC activation, EDS and postural orthostatic tachycardia syndrome (POTS) [75]. Moreover, there is an epidemiological link between POTS and chronic fatigue syndrome (CFS), as it was estimated that up to 15% of patients with CFS could meet the diagnostic criteria for POTS [76].

In addition, patients with EDS hypermobility type (the most prevalent type), have a higher prevalence of rheumatic diseases than the general population, including psoriasis, ankylosing spondylitis, rheumatoid arthritis and fibromyalgia. These patients also have a higher prevalence of the HLA-B27 antigen [77]. However, some authors have argued that the association of MC activation, EDS and POTS cannot be firmly asserted based on the currently available data, as it relies mostly on the presence of overlapping symptoms [73]. On the other hand, the link between MCs and CFS has been established more clearly, as it was shown that compared to healthy subjects, persons suffering from CFS have a higher number of peripheral MCs, that these cells have particular phenotypes, and that MC phenotypes correlate with the severity of the condition [78]. Long COVID is an emerging syndrome following the recent COVID-19 pandemic. In this context, it is noteworthy that patients suffering from long COVID claim symptoms resembling CFS, and MC activation symptoms were reportedly increased in long COVID [79]. High numbers of MCs are normally found in lung tissue and nasal mucosa. MCs are among the first immune cells to be activated in response to penetration of the SARS-CoV-2 virus [80]. However, the study conducted by Liu et al. should be mentioned, which suggests that MCs contribute to SARS-CoV-2 entry by generating the chymase/spike protein complex, highlighting a possible dual role of MCs in the pathology of COVID-19 [81]. 

Recent evidence suggests that in COVID-19, MCs play a crucial role in inducing inflammation by releasing pro-inflammatory cytokines, such as IL-6 and IL-1beta. These mediators can be released without degranulation and can be considered late-phase mediators. Activated MCs also contribute to COVID-19 brain fog by altering the permeability of the brain-blood barrier and by activating microglia [82]. Thus, some authors consider that COVID-19 brain fog suggests the activation of MCs [82]. Moreover, MCs are involved in the procoagulant state that characterizes COVID-19, through the release of VEGF, TNF-alpha and histamine [80] and stimulate fibroblasts, which leads to pulmonary fibrosis [83].

## 5. Mast Cells in the Skin

The skin, which is the largest immune organ [84], is also the tissue richest in MCs [11]. At this level, MCs play key roles: besides their well-known contribution to immediate-type allergic reactions, MCs are involved in the host protection against infection and toxins, in immunomodulation, regulation of the epidermal barrier function and skin homeostasis [85,86]. Furthermore, they are thought to contribute to wound healing and skin ageing [87,88]. 

At the skin level, MCs tend to accumulate around blood vessels, nerves, and hair follicles [87], with higher numbers located at the extremities (arms and legs) compared to proximal areas [89].

MCs contribute to skin homeostasis by interacting with keratinocytes (KCs) in an activating or inhibitory manner, depending on the type of MC-released mediators [90,91]. For example, keratinocyte growth factor [92] and platelet-activating factor [91,93] activate KCs, while histamine and heparin inhibit KC proliferation, in this way controlling epidermal regeneration [94,95].

By interacting with both blood and lymphatic vessel endothelial cells (ECs) via histamine, cytokines, leukotrienes, prostaglandins and growth factors, MCs can influence angiogenesis, and thus skin remodeling [22,96,97,98]. Angiogenesis is also promoted by MC-derived tryptase, through degradation of the basement membrane [99]. It is interesting that this interaction works both ways, with MCs also being a target for angiogenic factors [97,99]. For example, VEGF-A expressed by ECs can regulate MC proliferation and maturation within the skin [100].

Fibrosis and scar formation are correlated with the magnitude of the inflammatory response. The mast cell was proposed as an activator of fibroblasts, and thus responsible for the excessive collagen deposition. Numerous studies in animal and human models have indicated that MCs respond with degranulation to skin injury and that their number increases during repair as a result of recruited MC precursors from circulation [101,102,103]. It is also suggested that MCs play a part in every phase of the wound healing. In the early inflammatory phase, they are integral to infection prevention and recruit other immune cells, in the proliferative phase they stimulate angiogenesis and keratinocyte and fibroblast activity, and in the late (scar formation/remodeling) phase they play key roles through close communication with fibroblasts [87,104]. In the dermis, MCs are in close proximity to fibroblasts, which they stimulate via IL-4 [105], IL-13 [106], VEGF and FGF2 [107].

The MCs’ pro-fibrotic activity in the skin is supported by most published data, but recent studies bring conflicting information.

On the detrimental side, we can cite studies that found more MCs in scar tissues as opposed to normal skin [105,106,107,108], and studies that show scar mitigating therapies correlate with reduced MC numbers [108,109]. For example, a study in red Duroc pigs (used as a model of hypertrophic scarring) showed that collagen deposition and wound contraction reduced after treatment with ketotifen (a mast cell stabilizer), with the formation of collagen fibrils that appeared thinner and less dense [110].

However, in the case of keloid scarring, conflicting results have been reported, with some studies showing high histamine levels and elevated numbers of MCs present in keloid tissue [111], and more frequent allergic symptoms in keloid-forming patients compared to normal individuals [112], whilst other studies contradict these findings [113,114,115,116].

In humans, studies conducted in the case of systemic sclerosis patients [117,118] or dermal graft-versus-host disease [119] found higher numbers of MCs and higher frequency of degranulation compared to uninvolved skin/skin from unaffected controls.

## 6. MCAS

As discussed above, MC activation is associated with a significant number of clinical, and even subclinical, conditions and should be expected in any situation where local or systemic inflammation is triggered either acutely or chronically.

More than 10 years ago the term MC activation syndrome (MCAS) was proposed for severe systemic MC activation [120]. Since then, this relatively new nosologic entity has come to be viewed as the expression of “aberrant” MC activation [121], as opposed to diseases where MCs are in a greater number than normal, and a group of authors expanded the category of patients to include chronic, mild, but still multi-system symptoms of inflammatory/allergic nature. Some studies even describe a prevalence as high as 17% of the population [122], with 74% of the patients reporting first-degree relatives with similar symptoms.

Since mastocytosis is considered a rare condition, the above prevalence may be viewed as huge. Mastocytosis is a group of disorders characterized by clonal proliferation and accumulation of abnormal MCs in the skin and possibly other organs. Most of the genetic mutations reported to date involve the stem cell factor receptor c-KIT, present on the surface of MCs. The main categories of mastocytosis are cutaneous mastocytosis (CM)—MC proliferation limited to the skin—and systemic mastocytosis (SM)—at least one extracutaneous organ or system involved [123]. 

Discussing the heterogenous group of mastocytoses is beyond the scope of this review; however, it should be noted that, as we describe later, many of them satisfy the criteria for MCAS as presented by some of the mainstream authors.

### 6.1. MCAS Clinical Manifestations

MCAS patients usually present a plethora of non-specific complaints, and often associate important comorbid conditions [124]. One has to be prepared to consider their complaints real, before dismissing them as somatizations. The common symptoms are listed in Table 1, the most typical being flushing, hypotension (possibly leading to syncope), urticaria, angioedema, wheezing, headache, cramping, vomiting and diarrhea [123].

### 6.2. MCAS Diagnostic Criteria

The criteria for MCAS were established for the first time in 2012 [127], and continue to be refined by an international consensus group [15,128]. Although entities like “mast cell activation (disorder), unspecified”, “mast cell activation (syndrome)”, “monoclonal MCAS”, “idiopathic MCAS” and “secondary/reactive MCAS” were recently assigned codes in the United States International Classification of Diseases, Tenth Revision, Clinical Modification (ICD-10-CM) [128], there is still no certainty as to whether cases of MCAS are not missed versus the possibility of overdiagnosis.

**Figure 2:**
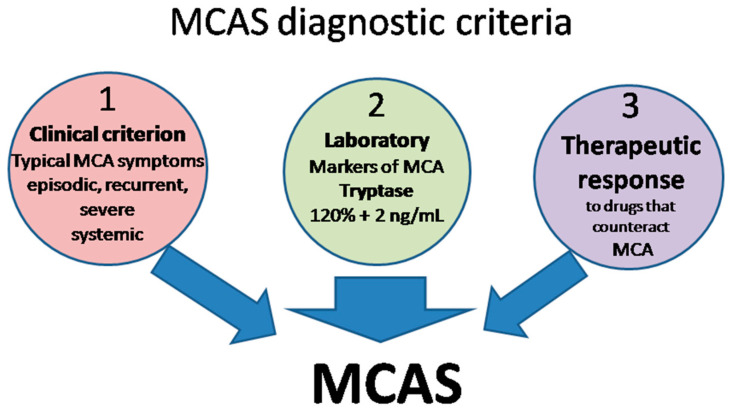
MCAS diagnostic criteria. MCA, mast cell activation; MCAS mast cell activation syndrome.

The definition of MCAS is based on three types of criteria (Figure 2) that all have to be met for an MCAS diagnosis to be established [15,120,127,129,130].

1. Clinical: typical MC activation symptoms, which are episodic, recurrent, severe (often taking the form of anaphylaxis) and systemic (involving two organ systems at least);
2. Laboratory: markers of MC activation—event-related serum tryptase level elevated above 120% of the individual’s serum baseline + 2 ng/mL;
3. Therapeutic: clinical response to drugs that counteract MC mediators or prevent their release.

**Clinical criterion**: symptoms of MCAS are multi-organ/system and extremely polymorphous, but only a small number of them are taken into consideration when establishing the diagnosis [125] (Table 1). For example neuro-psychiatric manifestations were recently excluded, although many patients listed “brain fog” [131] as one of their main complaints, together with stress as an important trigger [132,133]. The validated clinical criterion [15,120,127,129,130,134] requires episodic, recurrent, severe symptoms of MC activation that involve at least two organ/systems (e.g., flushing associated with hypotensive syncope). 

**Laboratory criterion**: requires an acute, event-related, increased level of a laboratory marker of systemic MCA. The standard marker is serum tryptase, and the normal serum level in adults is considered between 0 and 11.4 ng/mL. Event-related means that the sample must be obtained within a 1 to 4 h interval from the onset of symptoms and the basal level should be pre-assessed in a symptom-free interval, or after at least 24–48 h from complete recovery. However, some authors have asserted that normal tryptase levels do not exclude MCAS [126,135]. Other mediators, like histamine (plasma, urine), prostaglandin D2 (plasma), chromogranin-A (plasma), leukotriene E4 (urine) or mediator metabolites (N-methyl histamine and 1-methyl-4-imidazole acetic acid, which are urinary metabolites of histamine and of prostaglandin D2, respectively) do not yet have validated criteria for the degree of elevation required for an MCAS diagnostic, and are considered less specific for MC activation [124]. 

**Therapeutic criterion**: requires a documented response to drugs that specifically address MC activation such as antihistamines, leukotriene modifiers, MC stabilizers or cyclooxygenase inhibitors or biologics like omalizumab [15,120,125,127,129,134].

A validated questionnaire—Mast Cell Mediator Release Syndrome (MCMRS) [123]—can help diagnose a mast cell activation disorder (MCAD). It identifies the most common symptoms of MCA, orienting in this way the next diagnostic steps.

Of the above criteria, the clinical one is the most controversial, as there are authors holding the view that a majority of patients present chronic, mild to moderate ailments, and that these patients should not be excluded from the MCAS diagnosis [126]. In a combined MCAS retrospective/prospective analysis (298/115 patients, respectively) Afrin et al. [126] investigated symptoms, comorbidities, demographics, family histories, physical exams and laboratory data. The authors have found that the majority (69%) were female; median number of comorbidities was 11 (range 1–66); median number of symptoms/patient was 20 (range 2–84). Median time from symptom onset to diagnosis was 30 years (range 1–85). In the authors’ words, “although most of the patients (72%) appeared chronically ill at some point, in general the studied patients appeared healthier than would be expected from their litanies of complaints (e.g., presenting with an objectively normal-for-age extent of hair despite complaint of excessive hair loss), likely contributing to a primary psychiatric diagnosis (especially anxiety, depressive, somatoform, or conversion disorders) experienced by most at one or more points in their extensive prior evaluations”.

Therefore, some authors consider the current MCAS criteria excessively restrictive. For instance, the original criteria proposed by Molderings et al. [135] allowed for the diagnosis of a larger group of patients who would not be given a diagnosis by Valent et al. [128].

### 6.3. MCAS Classification

The most important classification criterion for MCAS is clonality (clonal vs. non-clonal). Clonal MC disturbances include the presence of c-KIT mutations (usually the D816V gain-of-function mutation, but not only) and/or expression of CD25, CD2 or CD30 on MCs [128]. If this is the case, MCAS is considered primary. In time, patients suffering from primary MCAS may develop overt SM. If MC activation is due to an allergic condition or another hypersensitivity disorder (IgE or non-IgE mediated, as for example drugs interacting with MRGPRX2) [136], then MCAS is classified as secondary (non-clonal). On the other hand, if there is no clonality, and no other specific cause can be identified, the MCAS is considered idiopathic. Combined forms of MCAS were also described, in which patients have traits of both primary and secondary MCAS (clonality + documented IgE-dependent allergy for example) and fall into the category of mixed MCAS [15].

MCAS differential diagnosis includes a large number of medical areas, conditions and disorders: infectious diseases (severe viral/bacterial/parasitic infections, septic shock, acute gastrointestinal infection), gastrointestinal (food intoxication, VIPoma, gastrinoma, irritable bowel syndrome, eosinophilic gastroenteritis or esophagitis, inflammatory bowel disease), cardiovascular (endocarditis or endomyocarditis, myocardial infarction, pulmonary embolism, aortic stenosis with syncope), endocrine (pheochromocytoma, carcinoid, medullary thyroid carcinoma), neuropsychiatric (anxiety/panic attacks, vasovagal syncope), cutaneous (different kinds of urticaria and angioedema, drug related pruritus/rashes, rosacea, vasculitis, atopic dermatitis). Furthermore, differential diagnosis should take into consideration two conditions where there is a chronic systemic elevation of MC mediators without MCs undue activation, namely histamine intolerance (HIT) and hereditary alpha tryptasemia (HαT) (see below). A complete physical examination, combined with a detailed patient history and laboratory assessment of specific markers, can help exclude these conditions [137].

### 6.4. Histamine Intolerance

Food intolerance (non-allergic food hypersensitivity) is an abnormal response of the organism, non-immunological in nature, to the ingestion of food in a dosage normally tolerated [138]. Histamine intolerance (HIT) groups the various manifestations of accumulated histamine (endogenous and exogenous) resulting from a degradation deficit (similar to lactose intolerance, where the lactase enzyme is lacking), in this case the deficiency is ascribed to the diamine oxidase enzyme (DAO) [139]. 

HIT has to be differentiated from histamine intoxication, occurring when large quantities of histamine are ingested, as in the case of scombroid poisoning [140]. 

Histamine, through its ubiquitously distributed receptors, can affect various organs and systems in many different ways, making it difficult for the clinician to establish a typical clinical picture. The description of symptoms is overlapping with much of the MCAS manifestations, except that they occur following food ingestion. Typical signs include flushing, pruritus, urticarial lesions and gastrointestinal manifestations (diarrhea/constipation, abdominal pain, bloating). Sometimes patients can experience tachycardia [141], low blood pressure, headaches, dizziness and respiratory symptoms like rhinorrhea, nasal congestion, sneezing [142]. The most confounding problem when attempting to diagnose HIT is that similar stimuli may lead to different manifestations in the same individual at different moments in time [143].

HIT is currently diagnosed using low serum level of DAO as a biochemical marker, with a proposed threshold at 10 U/mL [144]. In addition, two or more histamine intolerance symptoms, improvement with a low histamine diet and response to antihistamines are considered diagnostic criteria [145].

### 6.5. Hereditary Alpha Tryptasemia (HαT)

HαT is an autosomal dominant genetic trait that may be present in up 5% of the general population [146]. It is characterized by elevated serum basal tryptase (above 8 ng/mL), caused not by MC proliferation or activation, but as a result of increased baseline synthesis [146]. Patients have multiple copies of the TPSAB1 gene which encodes for α-tryptase [14]. As a result of these high levels of tryptase, MC activation-type symptoms can be generated: pruritus, flushing, urticaria/angioedema/anaphylaxis, rhinitis, asthma, irritable bowel syndrome and many other pulmonary, cardiovascular and neuropsychiatric disturbances [123]. An association between elevated tryptase levels and the severity of anaphylaxis induced by food or other causes was reported by several studies [147,148,149,150]. 

A recent study [151] involving patients suffering from SM, venom and idiopathic anaphylaxis coming from multiple international cohorts found that HαT is associated with an increased risk for anaphylaxis in the SM patients. Importantly, the authors showed that HαT is significantly more prevalent among the population presenting with idiopathic anaphylaxis and SM. HαT can coexist with MCAS and this further complicates and aggravates the clinical picture, increasing the risk for severe anaphylaxis [128]. In light of this, HαT is now considered the first described common heritable genetic modifier of anaphylaxis. Therefore, tryptase genotyping should be considered in any individual with a tryptase level ≥8 ng/mL, in order to assess the risk of HαT [15].

Wondering whether patients with hereditary alpha tryptasemia (HαT) and documented MCAS should be classified as suffering from primary MCAS, secondary MCAS or as idiopathic MCAS when no other underlying etiology or disease is detected, Valent et al. proposed another category which combines MCAS and hereditary alpha tryptasemia (HαT) [128].

### 6.6. Cutaneous Manifestations

Dermatologic symptoms were mentioned starting with the first cases described in the literature, which emerged in 2007, when Molderins et al. studied a cohort of 17 patients with MC activation disorder (MCAD) [152], including seven patients with SM and four patients not satisfying the SM criteria but presenting with an MCAS-like picture. In these four patients, dermatologic symptoms were prominent and consisted of flushing, anal pruritus, clotting dysfunction and unspecified “skin-signs”. In 2008, Butterfield and Weiler presented four patients with symptoms of MCA, in which they described pruritus, flushing, urticaria and angioedema [153].

Since these first mentions of non-proliferative MCAD, the published case reports and series studies have accumulated evidence of extremely polymorphous cutaneous manifestations including migratory pruritus [154], dermatographism [126,155,156,157], hives [153,158], waxing and waning diffuse migratory rash [154], edema/migratory edema [126], migratory abdominal wall cellulitis [155], xerostomia [155,158], telangiectasia, bleeding tendency [159], eczema, Reynaud’s syndrome [157] and diaphoresis [160].

In 2017, Afrin et al. [126] published a cross-sectional study on 413 patients and listed the following results: dermographism (76%), pruritus/urticaria (63%), edema (up to 56%), rash (up to 49%), sweats (up to 49%), bruising (up to 39%), flushing (up to 31%), poor healing (23%), alopecia (15%), pallor (13%) and onychodystrophy (13%).

A literature review published in 2019 [161] that included the aforementioned article in addition to 15 other iMCAS selected studies and totaling 562 patients, reports the following: flushing (34.9%), pruritus (32.2%), clotting/bleeding disfunction (28.2%) and urticaria/hives (14.1%). The review included cases meeting criteria that were not exactly the MCAS diagnostic criteria: (1) episodic symptoms suggesting MC activation, (2) increased markers (blood and urine) of MC activation, (3) no evidence of other causes (including other defined idiopathic entities) of MC activation. 

The MCAS diagnosis should be made after first ruling out the rest of MCADs, which are proliferative, involve atypical (immunohistochemically identifiable MCs), and have well-established diagnostic criteria [162]. The well-known cutaneous manifestations of mastocytosis include:

- Maculopapular cutaneous mastocytosis (urticaria pigmentosa)—hyperpigmented macules, papules or nodules usually on the trunk associating Darier’s sign (formation of a wheal in response to stroking/rubbing the skin), although the absence of it does not rule out the diagnosis [163];
- Diffuse cutaneous mastocytosis—a rare variant of cutaneous mastocytosis with onset usually at birth or in early infancy [164], manifesting in children with generalized erythema and thickened skin, variable pigmentation and in some cases with papules and intense dermographism [165]; in adults it presents with extensive bullae, that sometimes can be hemorrhagic, and which progress to erosions, desquamation and finally hyper-pigmentation and also intense dermographism [165]
- Mastocytoma of the skin—usually in children, presents at birth or during the first 3 months and resolves spontaneously in childhood [163]; appears as a single yellow or brown lesion (macule, plaque or nodule) or rarely 2–3 lesions, on the extremities [166].

Systemic mastocytosis associates systemic manifestations and the diagnostic has to be based, as in the case of cutaneous mastocytosis, on the World Health Organization (WHO) criteria [162]. 

A skin biopsy obtained from persistent lesions (lasting more than 24 h) is useful in order to exclude other possible pathologies such as the different types of mastocytoses (associated with tryptase immunohistochemistry), urticarial vasculitis/other vasculitides, auto-inflammatory urticarial syndromes, hypereosinophilic syndromes, urticarial dermatitis, prurigo, bullous pemphigoid and Sweet syndrome. 

After excluding mastocytosis, disorders with known mast cell involvement like acute/chronic urticaria (including inducible urticaria, chronic spontaneous urticaria with its sub-category of autoimmune urticaria) or mast cell-mediated angioedema should be considered. In this phase things get slightly more complicated, especially in the case of anaphylaxis, where by definition at least two organ systems must be affected [167]. MCAS diagnostic criteria are satisfied by any repetitive, systemic mast cell activation severe enough to raise the level of tryptase as required by the laboratory criterion [168]. Moreover, the possibility of classifying as secondary MCAS cases where MC activation occurs as a result of a definite cause, like Hymenoptera venom for example, confuses things even more. For example urticaria, when isolated, is considered by Valent et al. [128] as a form of local MC activation, not to be classified as MCAS. It appears that in this category of disorders, diagnosing MCAS is a question of degree of intensity and spread of inflammation.

Considering the diversity of cutaneous signs and symptoms of MCAS presented in the scientific literature published to date, a very long list of dermatological conditions has to be excluded. We present some of the alternative diagnoses for the main dermatologic presentations (usually included as clinical criteria) in Table 2 [125].

### 6.7. Treatment

For most patients with MC-associated symptoms a cure is not possible at present. The management of these patients includes identification and avoidance of triggers, medications that block mediator effects on tissues and/or mediator release, and in severe cases, drugs that reduce the number of MCs. In addition, the symptomatic treatment of specific complaints is useful. Management should be multidisciplinary and individualized, considering the complexity of most cases and patients’ unique constellation of symptoms, reactions to different triggers and response to treatment.

The list of triggers that can activate MCs is extensive and includes many everyday life substances (e.g., alcohol, cigarettes, chemicals including drugs, and high histamine foods) or situations (e.g., emotional stress, cold, heat, UV light, travel by car). Avoiding triggers is not only difficult, but often the patient (and therefore the doctor) is unaware of which of the substances contained in a product may be responsible for the symptoms. To help clarify personal susceptibilities patients are advised to keep a journal, while family members should also be educated. Lifestyle changes include adopting a low-histamine diet, including exercise in the daily routine, balancing diet with supplemental prebiotics and/or probiotics, reducing stress and getting enough sleep.

Patients with anaphylaxis should receive education on presenting signs and symptoms, methods of avoiding known triggers, and should be referred to an allergist/immunologist [167].

## 7. Pharmacological Treatment

1. An epinephrine autoinjector should be carried by all patients who had an anaphylaxis episode in their personal history—especially patients with known allergies to insect venoms—or who have a known risk of developing one. Epinephrine should be administered intramuscularly as soon as the first signs are noticed, or after insect bites and stings, in a dose of 0.01 mg/kg to a maximum of 0.5 mg in adults and 0.3 mg in children [167].
2. Antihistamines are the first line of treatment for patients with non-life-threatening symptoms. They act by binding to, and blocking histamine receptors in the target tissues, thus reducing the impact of MC degranulation. Decades of clinical practice have established that the use of antihistamines alleviates symptoms such as itching, pain, urticaria, and acid reflux [169]. If symptom control cannot be achieved with the highest recommended doses of H2 antihistamines, a combination of non-sedating H1 and H2 antihistamines can be attempted, especially if the patient associates gastric symptoms [170]. Most patients have benefited from long-term antihistamine use, with good tolerance and minimum side effects [170].
3. Leukotriene inhibitors are used in asthma and respiratory symptoms with probable underlying bronchoconstriction with allergic triggers that do not fulfil the criteria for asthma. They are also used in the treatment of allergic rhinitis, psoriasis and atopic dermatitis. Drugs with two mechanisms of action are currently approved: leukotriene receptor blockers (montelukast and zafirlukast) and 5-lipoxygenase inhibitors that block synthesis of leukotrienes from arachidonic acid (zileuton). Leukotriene inhibitors can be prescribed in combination with antihistamines [171,172,173].
4. MC stabilizers increase the threshold for MC degranulation signaling. Cromolyn sodium and nedocromil are currently approved. Although very promising on paper, these drugs are not more effective than antihistamines and leukotriene inhibitors, with possibly more significant side effects. Consequently, they are not routinely prescribed. Moreover, ketotifen (a second-generation H1 receptor antagonist with higher brain permeability compared with the newer second-generation antagonists desloratadine and levocetirizine) has MC stabilizer activity. Desloratadine and cetirizine have also been reported as MC stabilizers, although doses significantly higher than the ones currently used for therapy were required [174,175,176,177,178].
5. Steroids should only be used short term for acute exacerbations of symptoms not controlled by standard therapy. Potential uses include edema, urticaria, wheezing, diarrhea and severe pain [179].
6. Biologics: omalizumab, an IgE receptor blocker, effectively controls MC-mediated symptoms, improves the patients’ quality of life, and should be recommended as low-dose long-term therapy to persons at risk of anaphylaxis [180,181]. Emerging kinase inhibitors (imatinib, mitostaurine, avapritinib) inhibit MC proliferation and IgE-dependent MC activation. These drugs may represent future effective therapies for patients with advanced systemic mastocytosis [168].
7. DAO oral supplements should be considered in the case of HIT [182].
8. Natural components with suggested MC stabilizing properties may be useful in the management of certain patients. Examples include quercetin, luteolin, resveratrol and green tea [183,184,185,186].

## 8. Discussion

MCs are unique among the cellular members of the immune system, as the only non-circulating granulocytes [4]. They play key roles in host defense, inflammation and tissue repair [6]. In proportion to their proven importance in mammalian physiology, their dysregulation can have devastating effects. Aberrant, system-wide MC activation can kill the organism in minutes, by initiating the rapid cascade of anaphylaxis. The emotional impact of such an event on both the patient and on the doctor, has resulted in the fundamental physiological roles of MCs to be overlooked for decades. Fortunately, this situation has changed in recent years, and research, both fundamental and clinical, carried out by numerous groups around the world has established the multifaceted significance of these most denigrated cells [3]. 

To their importance attests the many ways in which MCs can be activated, their complex array of molecular mediators which have a context variable pattern, and the large number of cells with which they interact, both of immune and non-immune origin, all of which lead to considering MCs as orchestrators of the local defense [3]. Recent genetic data further emphasizes their significance. In particular, family aggregated variability in the TPSAB gene copy may correlate with MC activity. Importantly, the fact that as yet no individual/family has been identified with less than two TPSAB copies in their genome, together with MCs’ role conservation throughout the vertebrate subphylum, strongly indicates that MCs are essential for survival and homeostasis in the living environment [4,19].

In 1991 Margie Profet suggested that extensive MC activation, including one that may lead to anaphylaxis can be justified, if it is produced in response to a potentially catastrophic aggression, such as envenomation [187]. At the time when this hypothesis was first put forward it was generally ignored. Today it seems that 30 odd years later, the author may live up to her name, as recent data shows that the presence of specific IgEs with their consequent activation of MCs, can help an organism survive what would otherwise be a fatal dose of venom [188]. 

Mirroring their many roles in host defense, MC involvement in pathology is also vast. Of note, the least striking dysregulations may contribute more to morbidity than the unmissable ones. Most of us will never face a significant risk of anaphylaxis throughout our life, while nearly everyone will suffer at one time or another from some MC-mediated cutaneous or digestive reaction to an external aggression (infectious, physical, chemical, or allergic). For such reactions, the intricate workings of MC machinery may not necessarily need to be understood, if they are single events in one’s life. Such an instance, even if severe, can be resolved by symptomatic treatment. Indeed, even in the case of anaphylaxis the solution is symptomatic, and the difference between success and failure is usually produced by how promptly the treatment has been administered. However, if the condition is recurrent, or even worse chronic, the burden of the disease on the individual increases dramatically, as do the presentations to various specialists, hospital admissions and health care costs, while the patient’s quality of life is significantly affected.

There is still debate regarding the MCAS patient profile, the main question being whether MCAS should be reduced to a narrow range of severe, potentially life-threatening symptoms, or if the wide span of manifestations described by authors who allow for patients with mild, chronic polymorphic symptoms of systemic MC activation may also be included. Importantly, a prospective study for idiopathic MCAS showed that in only 2% of the patients with symptoms suggestive of MCAS the syndrome could be confirmed as per standard criteria, indicating that further research is urgently required in order to identify “the true underlying pathomechanism(s) in patients with suspected MCAS” [189]. For the dermatologists (familiar with anaphylaxis and its perils, and used to prescribe antihistamines, MC stabilizers, or glucocorticoids for a wide range of allergic/inflammatory conditions) becoming more aware of the latter group of patients might be of interest and would help improve their therapeutic attitude. Moreover, the idea that specific, identifiable, MC disfunctions (genetic/epigenetic in nature) might underlay some dermatological conditions considered to be idiopathic is worth taking into consideration. For example, there are gastroenterologists who support the idea that when associated with symptoms in at least one more organ system, irritable bowel syndrome might be a manifestation of idiopathic MCAS [145]. What about symptomatic dermographism or idiopathic urticaria accompanied by symptoms suggestive of MC activation in at least one other organ of the body? Future research will hopefully bring more answers.

Last but not least, the connection between MCAS and COVID-19 should be taken into consideration. An interesting observation is that the prevalence of MCAS (approximately 17%) is similar to the prevalence of severe cases of COVID-19. Thus, Afrin et al. suggest that the cytokine storm observed in severe forms of COVID-19 could be linked to an unusual reaction of dysfunctional MCs to the SARS-CoV-2 virus [190]. A recent study highlighted that active tryptase and IL-6 levels are higher in patients with post-acute sequelae of COVID-19 compared to patients with post-acute asymptomatic COVID-19, denoting MC activation [191]. MCAS offers valuable insights into the management of long COVID, as symptom improvement has been observed in patients with long COVID after the administration of MC-directed therapies (antihistamines, cromolyn, flavonoid, etc.) [79]. Identifying MCAS early in patients with long COVID could decrease systemic complications.

## 9. Conclusions

For patients suffering from various forms of MC dysregulation, MCAS classification represents a hope that their otherwise mysterious condition may be recognized and treated by their doctor. However, based on the current definition criteria an important proportion of patients may be left outside this relatively new clinical entity. International dialogue between all groups involved in treating these patients is essential for optimal case management.

## 10. Future Directions

Currently, the pathophysiology behind many conditions comprising the MCAS is not clearly understood. Consequently, in most cases the treatment cannot address the cause, and for many years patients might not even be recognized as potential MCAS sufferers. It is hoped that in the near future more markers of MC dysregulation, beyond serum tryptase will be standardized and will begin to be used in diagnosis. Furthermore, fundamental research might provide valuable insights into potentially divergent causes behind various diseases that can be ascribed to MCAS. In particular, genome-wide profiling of MCAS patients may reveal genetic patterns that predispose to this syndrome, as well as provide potential mechanistic clues and therapeutic targets.

## Figures and Tables

**Table 1:** Diagnostic and non-diagnostic symptoms of mast cell activation syndrome [125,126].

Organ/System	Diagnostic Symptoms	Non-Diagnostic Symptoms
Cutaneous	Flushing, pruritus, hives, angioedema	Dermographism, urticaria, rashes, edema/migratory edema, alopecia, poor healing, onychodystrophy
Respiratory	Shortness of breath, laryngeal edema, wheezing, hypoxia, nasal congestion, sneezing	Dyspnea, asthma, cough
Gastrointestinal	Vomiting, abdominal cramps, diarrhea	Constipation, alternating diarrhea/constipation, gastroesophageal reflux, dyspepsia, dysphagia, heartburn, nausea, elevated transaminases, hepatomegaly, multiple chemical/food sensitivity
Cardiovascular	Hypotension, syncope, collapse, incontinence	Chest pain, palpitations/dysrhythmias, systolic hypertension (mild/moderate), tachycardia
General		Fatigue, weight loss or gain, obesity, fever, chills, sweats, frequent or odd infections, heat and/or cold intolerance
Allergologic		Multiple/odd drug reactions
Neuro-psychiatric		Headaches, migraines, cognitive disfunction/brain fog, insomnia, anxiety, depression, dysautonomia symptoms (POTS), tremor, paresthesia
Musculoskeletal		Myalgia, arthralgia, joint hypermobility, fibromyalgia-type pain
Urogenital		Dysuria, frequency, urgency, prostatitis, polycystic ovarian syndrome, endometriosis
Ophthalmologic		Eye irritation, conjunctivitis, visual anomalies
Ears, nose, throat		Tinnitus, hearing loss, rhinitis, sinusitis, sore throat, oral irritation/sores
Hematological		Easy bleeding/bruising
Lymphatic system		Adenopathy/adenitis
Stomatological		Dental deterioration

**Table 2:** Possible alternative diagnoses associated with the symptoms considered as clinical criteria for cutaneous MCAS [125].

Flushing	fever, emotion, exercise, temperature changes, foods (spicy, fish—scombroid poisoning) or beverages (alcohol ingestion), medications (e.g., calcium channel blockers, nicotinic acid, disulfiram combined with alcohol) rosacea, climacteric flushing, carcinoid syndrome, pheochromocytoma, mastocytosis, anaphylaxis, thyroid—medullary carcinoma, VIPoma, renal cell carcinoma, neuro-psychiatric (anxiety disorders, Parkinson’s, multiple sclerosis, migraine, trigeminal nerve damage, Frey syndrome, Horner syndrome, Streeten syndrome, autonomic epilepsy, orthostatic hypotension), sarcoid, dumping syndrome, arsenic intoxication, basophilic granulocytic leukemia, malignant histiocytoma, idiopathic flushing
Pruritus	xerosis, scabies, noncutaneous causes (e.g., cholestatic/non-cholestatic hepatobiliary diseases, hyperthyroidism, uremic pruritus, myeloproliferative disorders such as Hodgkin/non-Hodgkin lymphoma, lymphocytic leukemia, polycythemia vera ± aquagenic pruritus, essential thrombocytosis, diabetes), psychogenic (depression, anxiety, obsessive-compulsive disorder, somatic symptom disorders, psychosis, substance use), subclinical mastocytosis, drug induced—either directly or via cholestasis (e.g., morphine-based analgesics, angiotensin-converting enzyme inhibitors, selective serotonin re-uptake inhibitors, nonsteroidal anti-inflammatory drugs can induce chronic pruritus without associated cutaneous lesions); for localized pruritus: spinal nerve compressions, notalgia paresthetica, small fiber neuropathy, parasitoses (e.g., enterobiasis)
Urticarial lesions	acute/chronic urticaria, urticarial vasculitis, urticarial dermatitis, urticaria multiforme, drug and viral exanthems, autoinflammatory syndromes (cryopyrin-associated periodic syndromes, Schnitzler syndrome, adult-onset Still disease, Gleich syndrome, neutrophilic urticarial dermatosis), hypereosinophilic syndromes, autoimmune progesterone dermatitis, polymorphic light eruption
Angioedema	bradykinin-mediated angioedema, hereditary angioedema, granulomatous cheilitis at an initial stage, eosinophilic cellulitis (Wells syndrome), allergic contact dermatitis (e.g., to hair dye), photoallergy
